# Unveiling Active Constituents and Potential Targets Related to the Hematinic Effect of Steamed *Panax notoginseng* Using Network Pharmacology Coupled With Multivariate Data Analyses

**DOI:** 10.3389/fphar.2018.01514

**Published:** 2019-01-08

**Authors:** Yin Xiong, Yupiao Hu, Lijuan Chen, Zejun Zhang, Yiming Zhang, Ming Niu, Xiuming Cui

**Affiliations:** ^1^Faculty of Life Science and Technology, Kunming University of Science and Technology, Kunming, China; ^2^Yunnan Key Laboratory of Panax Notoginseng, Kunming, China; ^3^Laboratory of Sustainable Utilization of Panax Notoginseng Resources, State Administration of Traditional Chinese Medicine, Kunming, China; ^4^China Military Institute of Chinese Materia Medica, 302 Military Hospital of China, Beijing, China

**Keywords:** steamed *Panax notoginseng*, hematinic effect, active constituents, mechanism, network pharmacology, multivariate data analyses

## Abstract

Steamed *Panax notoginseng* (SPN) has been used as a tonic to improve the blood deficiency syndrome (BDS) in the theory of traditional Chinese medicine. Here, we aim to unveil active constituents and potential targets related to the hematinic effect of SPN, which has not been answered before. In the study a constituent-target-disease network was constructed by combining the SPN-specific and anemia-specific target proteins with protein-protein interactions. And the network pharmacology was used to screen out the underlying targets and mechanisms of SPN treating anemia. Also, the multivariate data analyses were performed for the double screening. According to the results, 11 targets related to chemical constituents of SPN were found to be closely associated with the hematinic effect of SPN. Among them, the direct target protein of mitochondrial ferrochelatase (FECH) had the major role through the metabolic pathway. Meanwhile, Rk_3_ and 20(*S*)-Rg_3_ were predicted to be major constituents related to the hematinic effect of SPN by both multivariate data analyses and network pharmacology. And it was been validated by the pharmacologic tests that Rk_3_ and 20(*S*)-Rg_3_ could significantly increase the levels of blood routine parameters, FECH and its downstream protein of heme in mice with BDS. The study provides evidences for the mechanism understanding and drug development of SPN for the treatment of anemia.

## Introduction

Blood deficiency syndrome (BDS) is a common syndrome with high incidence in clinic of traditional Chinese medicine (TCM), which often occurs in patients with anemia. It is characterized by a decreased quantity of red blood cells (RBC) and white blood cells (WBC), usually accompanied by the diminished hemoglobin (Hb) levels or altered RBC morphology (Kassebaum et al., 2014). Patients with BDS are often accompanied with tachycardia, dizziness, shortness of breath, poor ability to exercise, and even loss of consciousness (Janz et al., 2013). The causes giving rise to BDS are similar to anemia, including the reduced erythropoiesis, excessive hemorrhage, and destruction of RBC, of which the last one mainly refers to hemolytic anemia (Tian et al., 2017). Hemolytic anemia can be induced by the administration of acetylphenylhydrazine (APH) and cyclophosphamide (CTX), which is also a classical model for BDS (Zhang et al., 2014). Today, hemolytic anemia is mainly treated with blood transfusion. This treatment is associated with mechanical shearing forces that accelerate red blood cell rupture, and lead to severe clinical complications, including intravascular hemolysis, tissue oxidative stress, and multi-organ dysfunctions (Baek et al., 2012). Therefore, the development of effective therapies or drugs with blood-enriching efficacy is beneficial for the prevention and treatment of the disease.

*Panax notoginseng* (PN) (Burk.) F. H. Chen, a well known medicinal herb in Asia, has been used to treat blood disorders for thousands of years (Li et al., 2017). In 1944, PN and the relative products were classified as dietary supplements according to the United States Dietary Supplement Health and Education Act (103rd Congress, 1994). Traditionally, PN has been used in both non-steamed and steamed forms. Unlike the non-steamed one treating bleeding and removing blood stasis, the steamed PN (SPN) is used as a tonic to enrich blood and tonify the body, which can improve the BDS by increasing the production of various blood cells in anemic conditions (Ge et al., 2015; Gu et al., 2015). In our previous study (Xiong et al., 2017b), we found that the treatment of SPN could reverse significantly the decrease of levels of WBC, RBC, Hb, and platelet (PLT) of anemic mice induced by APH and CTX, which were inapparent when treated with non-steamed PN. The result was consistent with the report from Zhou et al. (2014). This could be due to the variation in the chemical composition of PN during the steaming process (Wang et al., 2012a). Despite various studies on the processing methods, chemical components and bioeffects of PN (Lau et al., 2009; Sun et al., 2010), much less attention has been paid on its steamed form, let alone the specific active constituents and the underlying mechanisms related to the hematinic effect of SPN, which hinders the development and application of this valuable medicine.

Network pharmacology, as a system biology-based methodology, offers an effective approach to evaluate the pharmacological effects of herbal medicines at the molecular level by predicting the complex interactions of small molecules and proteins in a biological system (Tang et al., 2015; Li et al., 2017). It is considered to be a promising way to unveil properties of herbal medicines and provide valuable insights into current drug discovery and development. Compared with conventional “one target, one drug, one disease” mode, network pharmacology focuses on “multi-targets, multi-constituents treatment to diseases,” which coincides with the holistic and systematic concepts of TCM (Li et al., 2015; Quan et al., 2016). It was reported that network pharmacology has been applied to detect new pharmacologic effects of herbal medicines, to uncover the interactions between herbal compounds/formulas and complex syndrome systems, to determine the active constituents and their mechanisms of action (Tao et al., 2013; Sheng et al., 2014). Therefore, to better understand the molecular basis of the enriching-blood effect of SPN, we computationally recognized the active constituents and potential targets of SPN treating anemia by the network pharmacology approach coupled with multivariate data analyses, and experimentally validated the predicted results.

## Materials and Methods

### Computational Prediction of Hematinic Constituents and Targets of SPN Using Network Pharmacology Analyses

#### Database Construction

Based on the literature research and our previous works on chemical analysis of SPN (Sun et al., 2010; Xiong et al., 2017a,b), 20 compounds were selected, including ginsenosides of F_2_ (1), Rb_1_ (2), Rb_2_ (3), Rb_3_ (4), Rc (5), Rd (6), Re (7), Rg_1_ (8), Rh_2_ (9), Rh_4_ (10), Rk_3_ (11), 20(*R*)-Rg_2_ (12), 20(*S*)-Rg_2_ (13), 20(*R*)-Rg_3_ (14), 20(*S*)-Rg_3_ (15), 20(*R*)-Rh_1_ (16), and 20(*S*)-Rh_1_ (17); and notoginsenosides of C (18), R_1_ (19), and R_2_ (20) in SPN. The chemical structures of the composite compounds in SPN were obtained from TCM Database@Taiwan (TDT) or drawn with ChemDraw professional 15.0 (Chen, 2011). The targets of constituents were predicted by the online target prediction software of PharmMapper with a criterion of “fit score” >4^1^ (Wang et al., 2017). Gene and protein targets associated with the disease of anemia were collected from the Online Mendelian Inheritance in Man (OMIM) database (Amberger et al., 2015). Database of Interacting Proteins for protein-protein interactions (PPI) was employed to identify the possible interactions of the aforementioned targets. And all protein ID codes were converted to UniProt IDs (Wei et al., 2016).

#### Network Construction and Analysis

To provide the scientific and reasonable interpretation of the complex relationships between the constituents and targets associated with anemia, network analysis was performed. The chemical constituents, SPN putative targets, and anemia targets were all connected to construct a “constituent-target-disease” network with PPI information. Cytoscape 4.3 (Smoot et al., 2011) was applied to visualize and analyze the network, and calculate the topological features of each node in the network. Only the hub nodes (twofolds above the median “degree” value of all nodes) with higher values of “betweenness centrality” and “closeness centrality” (above the median value of all nodes) were identified as the candidate SPN targets for anemia.

#### Targets and Pathways Analyses

To unveil the mechanism of SPN treatment of anemia, DAVID Functional Annotation Bioinformatics Microarray Analysis^2^ was performed (Dennis et al., 2003) for the pathway enrichment analysis. The key target in the most significant enriched pathway was verified by performing *in vivo* experiment in the SPN treatment on anemia.

### Screening Hematinic Constituents of SPN Based on the Fingerprint-Effect Analyses

#### Chemicals

The reference standards of ginsenosides 20 (*S*)-Rg_3_ and Rk_3_ with a purity ≥ 98% were purchased from the National Institutes for the Control of Pharmaceutical and Biological Products (Beijing, China). Methyl alcohol and acetonitrile of HPLC grade were purchased from Sigma-Aldrich, Inc. (St. Louis, MO, United States). Ultrapure water was generated with an UPT-I-20T ultrapure water system (Chengdu Ultrapure Technology, Inc., Chengdu, Sichuan, China). APH was purchased from HuaXia Chemical Reagent Co., Ltd. (Chengdu, China). CTX was purchased from Xiya Chemical Industry Co., Ltd. (Shangdong, China). Mouse ferrochelatase (FECH) enzyme-linked immunoassay kit and heme enzyme-linked immunosorbent assay kit were purchased from Shanghai MLBIO Biotechnology Co., Ltd. (Shanghai, China). All other chemicals used were of analytical grade.

#### Sample Preparation

The preparation of SPN refers to our previous study (Xiong et al., 2017a). “Samples were obtained from a single batch of PN root in Yunnan, China. Steamed PN samples were prepared by steaming the crushed raw PN in an autoclave (Shanghai, China) for 2, 4, 6, 8, and 10 h at 105, 110, and 120°C, respectively. The steamed powder was then dried in a heating-air drying oven at about 45°C to constant weight, then powdered and sieved through a 40-mesh sieve.”

#### Animals

Animal experimental procedures in the study were strictly conformed to the Guide for the Care and Use of Laboratory Animals and related ethics regulations of Kunming University of Science and Technology. The protocol was approved by the Experimental Animal Welfare and Ethics Committee, Kunming University of Science and Technology. The experimental method refers to our previous study (Xiong et al., 2017a), that “Kunming mice, male and female, weighing 18–22 g, were purchased from TianQin Biotechnology Co., Ltd., Changsha, Hunan [SCXK (Xiang) 2014-0011]. Before the experiments, the mice were given one-week acclimation period in a laboratory at room temperature (20–25°C) and constant humidity (40–70%), and fed with standard rodent chow and tap water freely.”

#### HPLC Analyses

The sample solutions were prepared according to the method in our previous research (Xiong et al., 2017a). “HPLC analyses were done on an Agilent 1260 series system (Agilent Technologies, Santa Clara, CA, United States) consisting of a G1311B Pump, a G4212B diode array detector, and a G1329B autosampler. A Vision HT C_18_ column (250 mm × 4.6 mm, 5 μm) was adopted for the analyses. The mobile phase consisted of A (ultra pure water) and B (acetonitrile). The gradient mode was as follows: 0–20 min, 80% A; 20–45 min, 54% A; 45–55 min, 45% A; 55–60 min, 45% A; 60–65 min, 100% B; 65–70 min, 80% A; 70–90 min, 80% A. The flow rate was set at 1.0 ml/min. The detection wavelength was set at 203 nm. The column temperature was set at 30°C and sample volume was set at 10 μl.”

#### Blood Routine Test

210 km mice, half male and half female, were randomly divided into seven groups, namely the control group, model group, *Fufang E’jiao Jiang* (FEJ) group, and drug groups including raw PN (S1-S3), SPN at 105°C (S4-S8), SPN at 110°C (S9-S13), and SPN at 120°C (S14-S18), 10 mice in each group. The APH and CTX-induced anemia model was applied to evaluate the “blood enriching” function of PN combined with previous methods (He et al., 2015). The anemia model was established by intraperitoneal injected of CTX of 0.07 g/kg for the first 3 days and hypodermic injection of APH of 0.02 g/kg at the fourth day. Mice in the control group were administered with 0.9% normal saline, whereas other groups were administered with FEJ (8 ml/kg), and SPN samples at different steamed conditions (0.9 g/kg), respectively, by gavage for 12 days. Then the blood was collected for the routing analysis, including levels of WBC, RBC, Hb, and PLT after 30 min of the last administration. And the liver tissues were collected for the determination of FECH and heme levels.

#### Multivariate Data Analyses

##### Canonical correlation analysis (CCA)

Canonical correlation analysis is a multivariate analysis used to study the correlation between two sets of variables. It is used for the dimension reduction of PCA and to extract the main principal components, and then describes the whole linear relationship of two sets of variables by the relevance of two principal components (Shi et al., 2016). In our study, CCA was used to analyze the relevance between the peak area values from the HPLC fingerprints and blood parameters data.

##### Partial least squares regression (PLSR)

Partial least squares regression is performed to find the inner relationship between the independent variables (*X*) and dependent variables (*Y*), which are simultaneously modeled by taking into account *X* variance, and the covariance between *X* and Y (Martens and Naes, 1991). In our study, the *X* matrix is composed of the enhanced fingerprints, and the *Y* vector is constructed with the reference values of hematinic effect obtained by measuring the levels of WBC, RBC, Hb, and PLT. Then, *X* and *Y* are decomposed in a product of another two matrices of scores and loadings; as described by the following equations:

(1) $$X\;=\;TP^{T}+E$$

(2) $$Y\;=\;UQ^{T}+F$$

Where *TP^T^* approximates to the chromatographic data and *UQ^T^* to the true *Y* values; notice that the relationship between *T* and *U* scores is a summary of the relationship between *X* and *Y*. The terms *E* and *F* from the equations are error matrices. Hence, the PLS algorithm attempts to find latent variables that maximize the amount of variation explained in *X* that is relevant for predicting *Y*; i.e., capture variance and achieve correlation (Sundberg, 2008).

### Experimental Validation for the Predicted Results

#### Validation for the Screened Active Constituents

Peaks in the HPLC profile predicted to be responsible for the hematinic activity of SPN were then identified by reference standards, of which the activities were finally verified by pharmacologic evaluation using the methods described in the section of “Blood routine test.” 90 km mice, half male and half female, were randomly divided into seven groups, namely the control group, model group, FEJ group, and drug groups [including low, moderate, and high-dose ginsenoside 20(*S*)-Rg_3_ group; and low, moderate, and high-dose ginsenoside Rk_3_ group], 10 mice in each group. Mice in the control group were intraperitoneal injected with 0.9% normal saline, whereas other groups were intraperitoneal injected with FEJ (8 ml/kg), 20(*S*)-Rg_3_ (2.5, 5, and 10 mg/kg, respectively), and Rk_3_ (2.5, 5, and 10 mg/kg, respectively), respectively.

#### Validation for the Predicted Targets and Its Downstream Protein

The livers of mice from different groups were removed to detect the levels of FECH and heme. The livers of different groups of mice were homogenized with a homogenizer and centrifuged for 20 min to collect the supernatant. Mouse FECH enzyme-linked immunosorbent assay kit and mouse heme enzyme-linked immunosorbent assay kit were used to detect the levels of FECH and heme in the supernatant.

### Statistical Analyses

All data were expressed as means ± SD. SPSS 21.0 software (Statistical Program for Social Sciences, SPSS Inc., United States) was applied to carry out the two-tailed unpaired *t*-test. Umetrics SIMCA-P 11.5 software (Sartorius Stedim Biotech, Sweden) was applied for PLSR analysis. CCA was performed using MATLAB 7.0 (Matrix Laboratory, United States). A value of *P* < 0.05 was considered to be significant difference. A value of *P* < 0.01 was considered to be highly significant difference. EC_50_ value was fitted by probit regression with Origin 7.5 software for windows (OriginLab Corporation, United States).

## Results

### Computational Prediction Using Network Pharmacology Analyses

#### Constituents and Targets Prediction

On the basis of database construction, 203 putative targets with “fit score” >4 were predicted by PharmMapper for 14 compounds and 90 candidate protein targets associated with anemia therapy were collected by keyword-based searching over the OMIM database, which included 19 common targets out of SPN constituents and anemia. Therefore, ginsenosides 20 (*R*)-Rg_2_, 20 (*S*)-Rg_2_, Rb_3_, Rb_1_, F_2_, and Rc were eliminated due to their low binding affinity to all the candidate targets.

#### Network Construction

**FIGURE 1:**
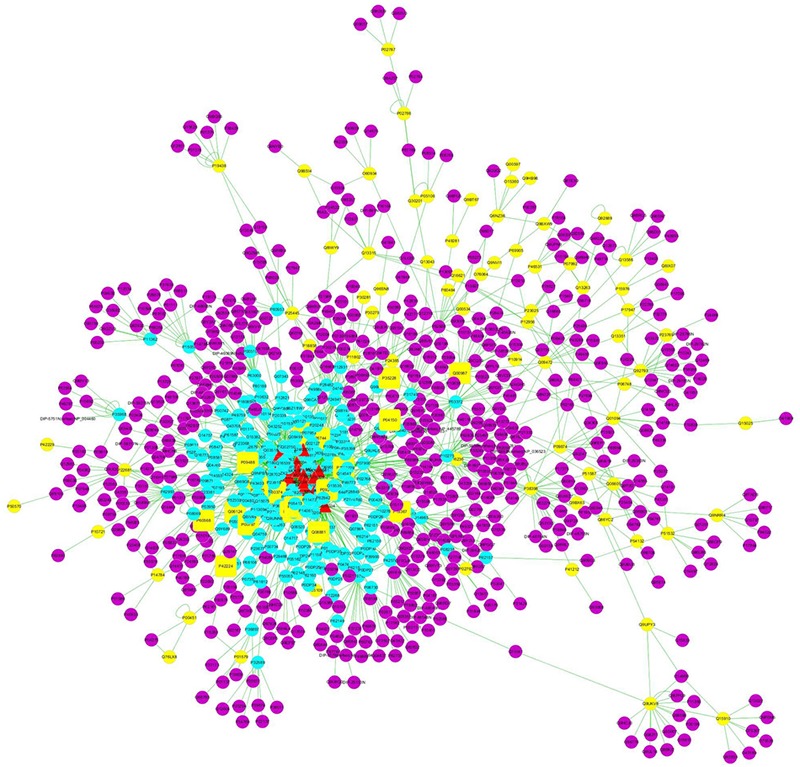
The “constituent–target–disease” network for the treatment of anemia with SPN. The red triangles represented the analyzed constituents of SPN, the blue dots represented the indirect targets of those constituents, the yellow dots represented the targets of anemia, the purple dots represented the interactional proteins of the anemia targets and SPN constituents, and the yellow squares represented the common targets of SPN constituents and anemia. SPN, steamed *Panax notoginseng*.

The “constituent-target-disease” network was constructed and the noteworthy features of the network analyzed could provide some important information for us to understand the “drug–target” interaction mechanism of a certain drug on a specific disease. Our study was focused on the effect of SPN on treating anemia. In Figure 1, the network for the constituents and their potential targets was illustrated with color-coded nodes. The intermolecular interactions (constituent-target or target- disease interactions) were indicated as links, i.e., edges between nodes (Tao et al., 2013). The red triangles represented the analyzed constituents of SPN, the blue dots represented the indirect targets of those constituents, the yellow dots represented the targets of anemia, the purple dots represented the interactional proteins of the anemia targets and SPN constituents, and the yellow squares represented the common targets of SPN constituents and anemia. Obviously, the common targets, as the directed targets of SPN on treating anemia, were relatively important for further screening.

Based on the network analysis, three topological parameters of “degree,” “betweenness centrality,” and “closeness centrality” were chosen to screen the potential anemia targets that SPN might affect. After calculating the values of the three parameters for each significant protein in the PPI network, the median values of “degree,” “betweenness centrality,” and “closeness centrality” were 1, 0, and 0.2183, respectively. The protein targets of which the “degree” was more than twofolds of the median value, and “betweenness centrality” and “closeness centrality” were higher than the median value, were chosen as the major targets of SPN treating anemia (Wang et al., 2018). As shown in Table 1, we finally determined that 11 common protein targets with degree ≧2, betweenness centrality ≧0, and closeness centrality ≧0.2183 for anemia therapy.

**Table 1:** The information of common target proteins and their corresponding active constituents predicted by network pharmacology analyses.

UniProt No.	Protein name	Closeness centrality	Degree	Betweenness centrality	Constituent
P22830	FECH, mitochondrial	0.2810	9	0.0011	(10) (11) (14) (15) (16) (17) (20)
P16442	Histo-blood group ABO system transferase	0.2995	16	0.0041	(3) (6) (7) (9) (10) (11) (14) (15) (17) (18) (19) (20)
P06702	Protein S100-A9	0.2811	8	0.0036	(3) (6) (7) (8) (10) (11) (17) (19)
P60568	Interleukin-2	0.2404	4	0.0056	(3) (14)
P06744	Glucose-6-phosphate isomerase	0.2490	2	0.0001	(20)
P30613	Pyruvate kinase PKLR	0.2747	4	0.0014	(7) (20)
Q06124	Tyrosine-protein phosphatase non-receptor type 11	0.2812	6	0.0078	(6) (10) (11)
P19367	Hexokinase-1	0.2635	2	0.0005	(7)
P00374	Dihydrofolate reductase	0.2677	2	0.0006	(7)
P35228	Nitric oxide synthase, inducible	0.2552	4	0.0114	(7)
P42224	Signal transducer and activator of transcription 1-alpha/beta	0.2188	2	0.0024	(18)

#### Pathway Analysis of SPN Treating Anemia

**FIGURE 2:**
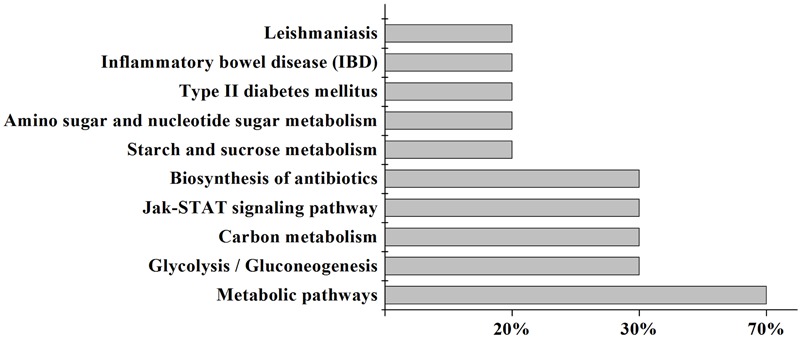
Ten enriched pathways with putative targets. *X*-axis represents the percentage of targets involved in the pathway. *Y*-axis represents the name of putative pathway.

**FIGURE 3:**
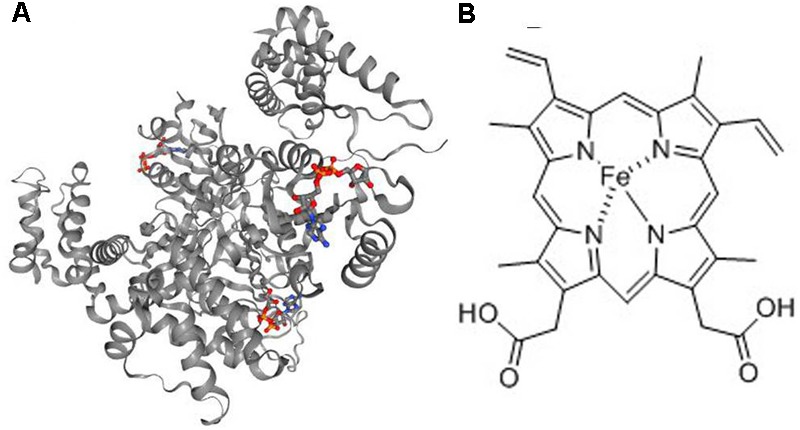
The structures of **(A)** FECH and **(B)** heme. FECH, ferrochelatase.

Kyoto Encyclopedia of Genes and Genomes (KEGG) is a knowledge base for systematic analysis of gene functions in terms of the networks of genes and molecules. The major component of KEGG is the pathway database that consists of graphical diagrams of biochemical pathways including most of the known metabolic pathways and some of the known regulatory pathways (Kanehisa and Goto, 2000). As shown in Figure 2, 10 KEGG pathways were enriched by the pathway-enrichment analysis. And there was the maximum quantity of targets involved in the metabolic pathways. Based on the analysis of KEGG, these target proteins were P22830 (FECH, mitochondrial), P16442 (histo-blood group ABO system transferase), P00374 (dihydrofolate, reductase), P06744 (glucose-6-phosphate isomerase), P19367 (hexokinase-1), P35228 (nitric oxide synthase, inducible), and P30613 (pyruvate kinase PKLR). Combined with the results in Table 1, FECH and histo-blood group ABO system transferase showed higher values of degree than others. And based on the literature research, FECH was reported to be a mitochondrial membrane-associated protein catalyzing the terminal step of heme biosynthesis (Yoon and Cowan, 2004), of which the abnormal synthesis can lead to anemia (Iolascon et al., 2009). Therefore, the protein of FECH and its downstream protein of heme (Figure 3) were chosen from the map of metabolic pathways for further verification. Constituents related to the target of FECH included ginsenosides Rh_4_ (10), Rk_3_ (11), 20(*R*)-Rg_3_ (14), 20(*S*)-Rg_3_ (15), 20(*R*)-Rh_1_ (16), 20(*S*)-Rh_1_ (17), and notoginsenoside R_2_ (20).

### Prediction of Hematinic Constituents of SPN Based on Multivariate Data Analyses

#### HPLC Analyses

**FIGURE 4:**
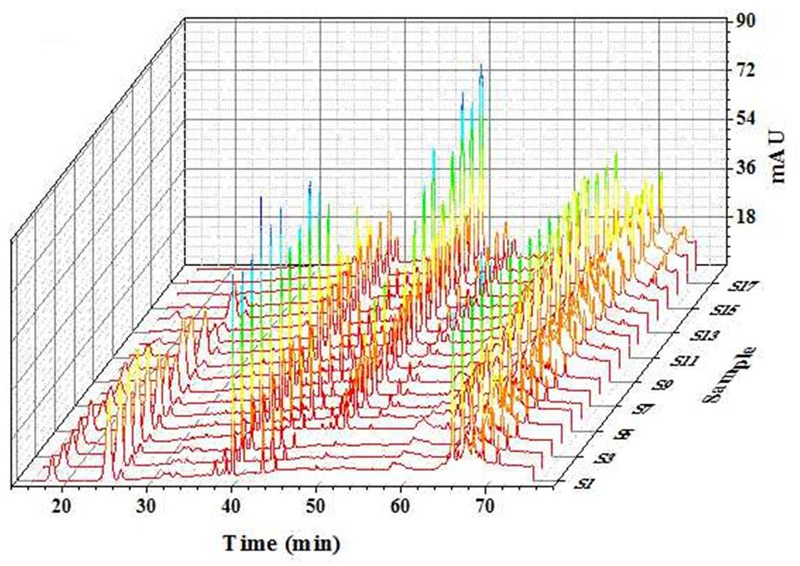
HPLC fingerprints of 18 batches of PN extracts. HPLC analyses were done on a Vision HT C_18_ column (250 mm × 4.6 mm, 5 μm) at 30°C. The mobile phase consisting of A (ultra pure water) and B (acetonitrile) was used at a flow rate of 1.0 ml/min as the following gradient mode: 0–20 min, 80% A; 20–45 min, 54% A; 45–55 min, 45% A; 55–60 min, 45% A; 60–65 min, 100% B; 65–70 min, 80% A; and 70–90 min, 80% A. The detection wavelength was set at 203 nm and the injection column was set at 10 μl. PN, *Panax notoginseng* (Xiong et al., 2017a).

HPLC fingerprints for 18 batches of PN samples were shown in Figure 4 (Xiong et al., 2017a). “Peaks with good segregation, which also occupied large areas from consecutive peaks, were determined as the common peaks of PN samples. Therefore, fifteen peaks were selected by comparing their ultraviolet spectra and HPLC retention time.” The areas of 15 peaks in 18 batches of PN samples were listed in Supplementary Table S1. “The peak area was defined as 0 for peaks lacked in chromatograms. The coefficients of variance for almost all common peaks were higher than 46.6%. This is due to the diversity in the levels of constituents contained in samples under different process conditions. The areas of 15 common peaks were used for the following analysis” (Xiong et al., 2017a).

#### Blood Routine Test

**FIGURE 5:**
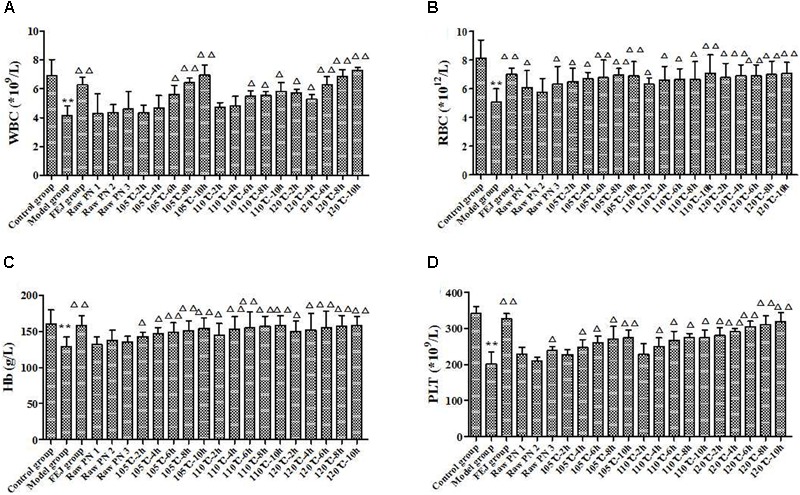
The contents of **(A)** WBC, **(B)** RBC, **(C)** Hb, and **(D)** PLT in the blood of mice after treated with different PN samples. Each value represents means ± SD (*n* = 10). ^∗∗^*P* < 0.01, compared with the control group; ^△^ *P* < 0.05 and ^△ △^ *P* < 0.01, compared with the model group. FEJ, *Fufang E’jiao Jiang*; Hb, hemoglobin; PLT, platelet; PN, *Panax notoginseng*; RBC, red blood cell; SD, standard deviation; WBC, white blood cell.

After the administration for 15 days, the quantities of WBC, RBC, Hb and PLT from the peripheral blood of mice were shown in Figure 5. Compared with the control group, the levels of WBC, RBC, Hb, and PLT in the model group were significantly decreased (*P* < 0.01), indicating the anemia model was successfully established. Compared with the model group, WBC, RBC, Hb, and PLT levels in the FEJ and all of PN groups were increased at different degrees. Besides, there were more significant differences in the levels of the above four parameters between the model group and SPN groups steamed at higher temperature and longer time, suggesting that SPN steamed at higher temperature and longer time could significantly reverse the decrease of the quantities of WBC, RBC, Hb, and PLT. While for mice treated with raw PN, the level of RBC was significantly increased, whereas there was no significant difference in levels of WBC, Hb, and PLT between raw PN and the model group, indicating that the blood-enriching effect of raw PN was generally weaker than SPN. According the results, SPN was observed to enhance the hematopoietic effect on mice with chemotherapy-induced anemia, which was consistent with the traditional use of SPN (Gu et al., 2015).

#### Uncovering Active Constituents by Multivariate Data Analyses

##### CCA

Canonical correlation analysis was used to establish the fingerprint-effect relationships between area values of 15 common peaks in the HPLC data and four blood routine parameters (WBC, RBC, Hb, and PLT). The analysis result was shown in Table 2. The correlation coefficients showed that the four parameters were positively correlated with *X*_4_, *X*_5_, *X*_9_, *X*_10_, *X*_11_, *X*_12_, *X*_13_, *X*_14_, and *X*_15_. Besides, eight peaks: *X*_5_, *X*_9_, *X*_10_, *X*_11_, *X*_12_, *X*_13_, *X*_14_, and *X*_15_ were highly correlated (| *R*| > 0.6) with the blood parameters. This indicates that the decrease of the quantities of WBC, RBC, Hb, and PLT might be reversed by these compounds.

**Table 2:** The correlation coefficients between the common characteristic peaks and four blood parameters.

Blood parameters	Common characteristic peaks															
	1	2	3	4	5	6	7	8	9	10	11	12	13	14	15
WBC	-0.689	-0.789	-0.595	0.122	0.5932	-0.590	-0.662	-0.274	0.740	0.782	0.783	0.805	0.738	0.684	0.747
RBC	-0.702	-0.777	-0.601	0.338	0.7276	-0.442	-0.611	-0.181	0.798	0.785	0.782	0.763	0.753	0.644	0.744
Hb	-0.854	-0.845	-0.712	0.156	0.757	-0.653	-0.748	-0.170	0.824	0.867	0.862	0.845	0.790	0.671	0.788
PLT	-0.845	-0.934	-0.776	0.212	0.7195	-0.676	-0.781	-0.479	0.905	0.940	0.939	0.909	0.907	0.886	0.933

##### PLSR

**FIGURE 6:**
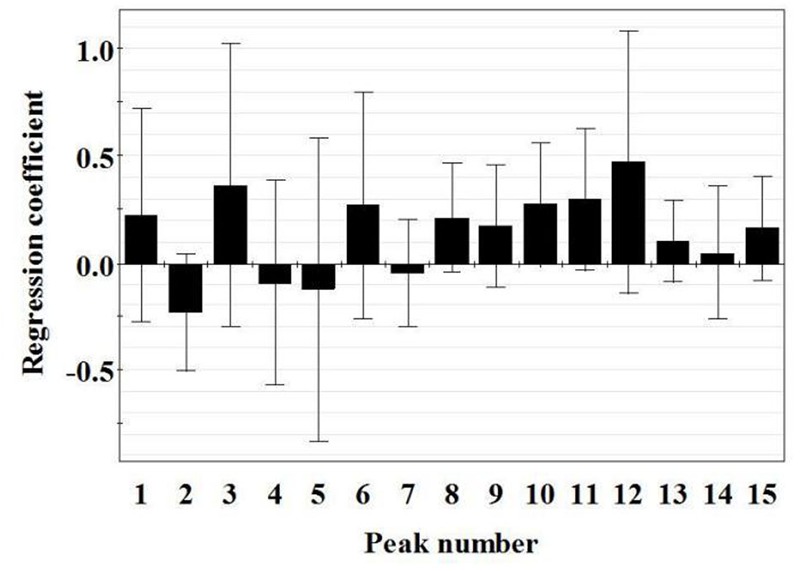
Regression coefficient plots of hematinic effect of 15 peaks in the chromatograms of PN samples. PN, *Panax notoginseng*.

The PLSR models to correlate chromatographic data and hematinic effect of 18 batches of PN samples were constructed. Since the total number of samples (18) was small and the prediction for new samples was not our first concern, no division was made into a calibration set to build a PLSR model and a test set to validate the predictive properties. Our main concern was to focus on the indication of hematinic effect peaks from the modeling results. PLSR models were built from the normalized data matrix *X* containing the 18 PN fingerprints and the response matrix *Y* (including *Y*_1_, *Y*_2_, *Y*_3_, and *Y*_4_, which represented WBC, RBC, Hb, and PLT, respectively). For the model, four principle components were achieved, accounting for an explained variance of 86.4% for *X* variable, 87.1% for *Y* variable, and a predictive ability (*Q^2^*) of 81.5% (Supplementary Table S2), indicating that the obtained model was excellent. As shown in the regression coefficients plot (Figure 6), peaks 1, 3, 6, and 8–15 were positively correlated with the quantities of WBC, RBC, Hb, and PLT, whereas peaks 2, 4, 5, and 7 were negatively correlated with the quantities of the four parameters.

Besides, the importance of the *X*-variables for the model could be summarized by variable importance for the projection (VIP) values (usually with a threshold >1.0). Thus, constituents corresponding to peaks 5, 9, 11, 12, 14, and 15, of which the VIP values were >1.0 (Table 3) with high absolute values of coefficients were considered to be highly related to the hematinic effect of different PN samples. Furthermore, false discovery rate (FDR, usually with a threshold ≤0.05) can effectively solve the control of false positive error in multiple comparisons of high-dimensional data, and can significantly improve the efficiency of hypothesis testing (Benjamini and Hochberg, 1995). Therefore, constituents corresponding to peaks 5, 7, 9, 10, and 12, of which the FDR values were ≤ 0.05, indicated that constituents corresponding to peaks 5, 7, 9, 10, and 12 were positively correlated with the hematinic effect. Among them, only peak 10 and 12 had significant correlation with the hematinic effect by *P*-value correcting (*P* < 0.05).

**Table 3:** Variable importance for the projection of PLSR.

Peak	1	2	3	4	5	6	7	8	9	10	11	12	13	14	15
VIP	0.987	0.70	0.56	0.29	1.03	0.37	0.57	0.45	1.121	1.37	1.24	1.41	0.902	1.09	1.23
FDR	0.097	0.085	0.092	0.088	0.041	0.067	0.034	0.068	0.042	0.048	0.072	0.022	0.099	0.061	0.060
*P*-value	0.091	0.097	0.098	0.558	0.055	0.150 0.0.235	0.185	0.235	0.079	0.011	0.062	0.001	0.086	0.079	0.058

##### Identification of active constituents corresponding to predicted peaks

**FIGURE 7:**
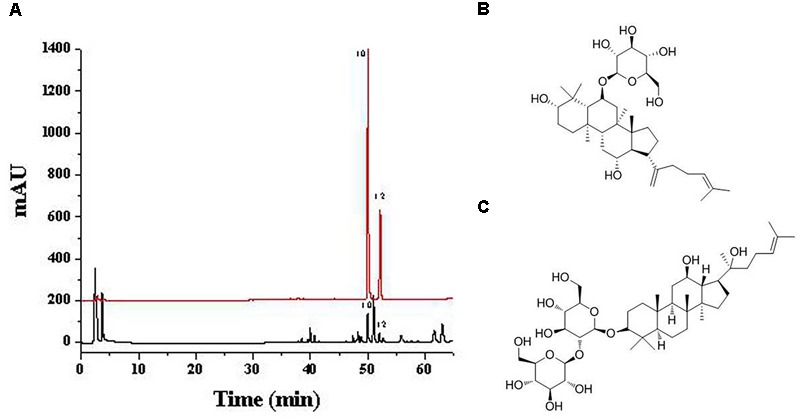
The **(A)** chromatograms of SPN sample (black) and the mix standard solution (red), and structures of **(B)** ginsenosides Rk_3_ and **(C)** 20(*S*)-Rg_3_. Peak 10 and 12 correspond to ginsenosides Rk_3_ and 20(*S*)-Rg_3_, respectively. SPN, steamed *Panax notoginseng*.

Based on CCA and PLSR results, constituents corresponding to peaks 10 and 12 were predicted to be the major active ones related to the hematinic effect of SPN. By comparing the chromatogram of SPN sample to that of the mixed standard solution (Figure 7), peaks 10 and 12 were identified to be ginsenosides Rk_3_ and 20(*S*)-Rg_3_, respectively, which had the major role in the hematinic effect of SPN.

According to the results of network pharmacology analyses, the two constituents were also predicted to be the active ones. Therefore, ginsenosides Rk_3_ and 20(*S*)-Rg_3_ were determined to be the target constituents for the further investigation of their hematinic effect.

### Experimental Validation for the Predicted Results

#### Validation for the Screened Active Constituents

**FIGURE 8:**
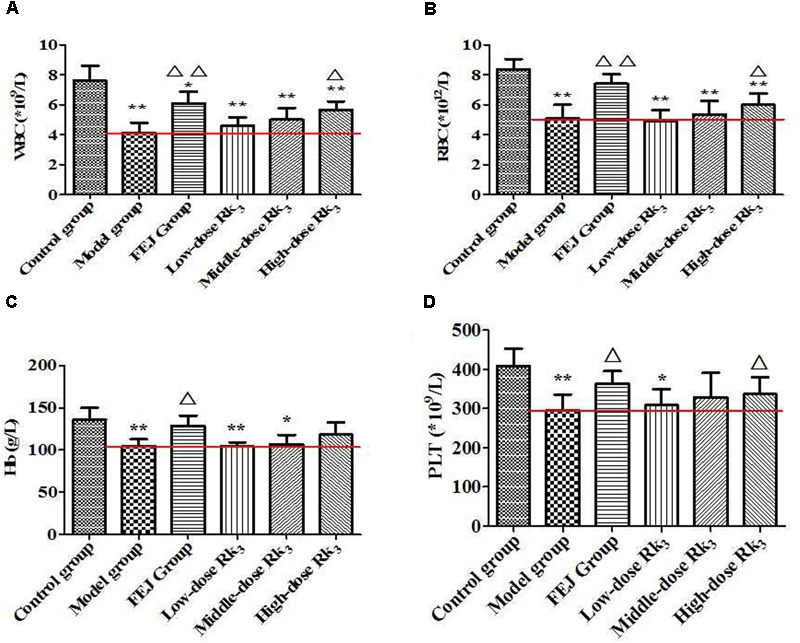
The contents of **(A)** WBC, **(B)** RBC, **(C)** Hb, and **(D)** PLT in the blood of mice after treated with ginsenoside Rk_3_. Each value represents means ± SD (*n* = 10). ^∗^*P* < 0.05 and ^∗∗^*P* < 0.01, compared with the control group; ^△^ *P* < 0.05 and ^△ △^ *P* < 0.01, compared with the model group. FEJ, *Fufang E’jiao Jiang*; Hb, hemoglobin; PLT, platelet; RBC, red blood cell; SD, standard deviation; WBC, white blood cell.

**FIGURE 9:**
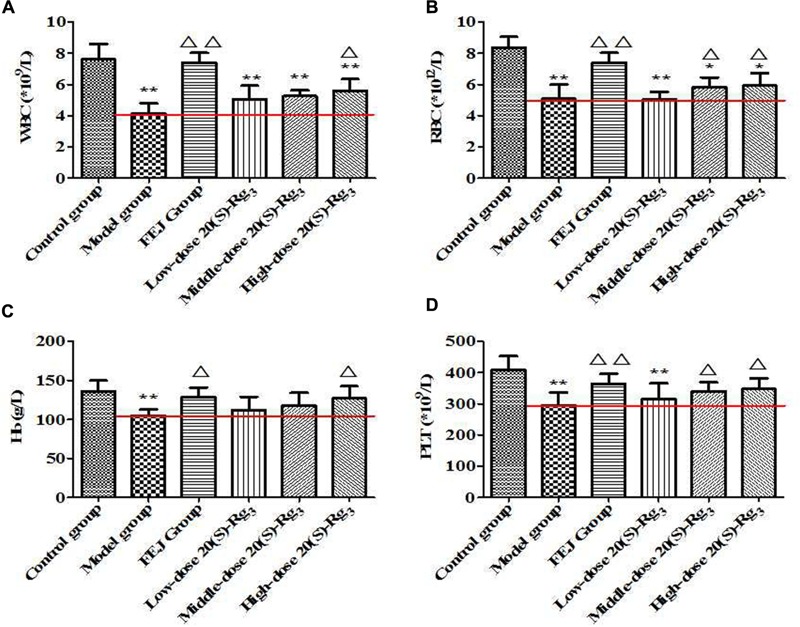
The contents of **(A)** WBC, **(B)** RBC, **(C)** Hb, and **(D)** PLT in the blood of mice after treated with ginsenoside 20(*S*)-Rg_3_. Each value represents means ± SD (*n* = 10). ^∗^*P* < 0.05 and ^∗∗^*P* < 0.01, compared with the control group; ^△^ *P* < 0.05 and ^△ △^ *P* < 0.01, compared with the model group. FEJ, *Fufang E’jiao Jiang*; Hb, hemoglobin; PLT, platelet; RBC, red blood cell; SD, standard deviation; WBC, white blood cell.

After intraperitoneal injected ginsenosides Rk_3_ and 20(*S*)-Rg_3_ for 15 days, the quantities of WBC, RBC, Hb, and PLT from the peripheral blood of mice were shown in Figures 8, 9, respectively. Compared with the control group, the levels of WBC, RBC, Hb, and PLT in the model group were significantly decreased (*P* < 0.01), indicating the anemia model was successfully established. Compared with the model group, the levels of WBC, RBC, Hb, and PLT after treated three doses of ginsenoside Rk_3_ were all increased. Meanwhile, the levels of WBC, RBC, and PLT in the high-dose group were significantly increased (*P* < 0.05), suggesting that Rk_3_ could reverse the decrease of the quantities of WBC, RBC, Hb, and PLT in a dose-dependent way. For the treatment with ginsenoside 20(*S*)-Rg_3_ of three doses, the levels of WBC, RBC, Hb, and PLT were all increased compared with the model group. Besides, the levels of WBC and Hb in the moderate-dose and high-dose groups were significantly increased (*P* < 0.05); and the levels of RBC and PLT in the high-dose group were significantly increased (*P* < 0.05), suggesting that the ginsenoside 20(*S*)-Rg_3_ could reverse the decrease of the quantities of WBC, RBC, Hb, and PLT in a dose-dependent way.

#### Validation for the Predicted Target Proteins

**FIGURE 10:**
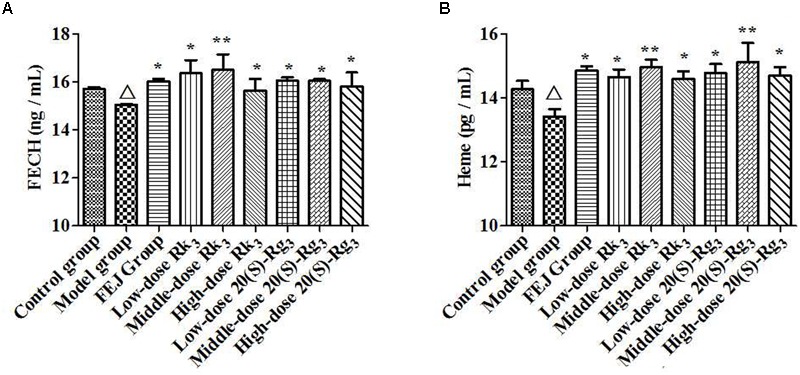
The contents of **(A)** FECH and **(B)** heme in the livers of mice. Each value represents means ± SD (*n* = 10). ^△^ *P* < 0.05, compared with the control group; ^∗^*P* < 0.05 and ^∗∗^*P* < 0.01, compared with the model group. FECH, ferrochelatase; FEJ, *Fufang E’jiao Jiang*; SD, standard deviation.

As shown in Figure 10, the level of FECH in the model group was significantly decreased compared with the control group (*P* < 0.05), suggesting that the model was well established. Compared with the model group, the levels of FECH and heme were all significantly increased after the administration of different doses of Rk_3_ and 20(*S*)-Rg_3_ (*P* < 0.05). In general, the levels of FECH and heme in livers of mice treated with the middle-dose Rk_3_ and 20(*S*)-Rg_3_ were relatively higher than those treated with low and high doses of drugs. The results indicated that ginsenosides Rk_3_ and 20(*S*)-Rg_3_ had a positive effect on improving the levels of FECH and heme, which was consistent with the predicted results of network pharmacology analyses.

## Discussion

Herbal medicines exert their therapeutic effects through the synergistic effects of multiple constituents and targets. PN in raw and steamed forms are historically supposed to be different in the efficacies. Lau et al. (2009) reported that the bleeding time of rats treated with raw PN was shorter than those treated with SPN. Zhou et al. (2014) found that SPN could significantly increase the levels of Hb and WBC, as well as the organ index of mice with BDS induced by CTX, which were unconspicuous when treated with raw PN. Based on our previous studies (Xiong et al., 2017b), there was a significant variation in the chemical composition between the two forms of PN, which leaded to the difference in the pharmacologic effects of raw and steamed PN. As shown in Figure 3, the levels of blood routine parameters of mice treated with SPN were significantly increased compared with the model group, which were also obviously higher than those of mice treated with raw PN. The result was consistent with the traditional use of SPN as a tonic to enrich the blood.

Currently, methods for uncovering active constituents of herbal medicines treating diseases mainly rely on retrospective analyses. However, this method depends on large consumption of manpower and material resources, which hinders the development of drugs. To address this issue, we have developed firstly a more comprehensive approach that integrates anemia-SPN networks to effectively discover potential active constituents and targets involved. Technically, the prediction accuracy of the drug targets and the completeness of the databases are important to the method and will affect the creditability of the final results. Therefore, we tried to reduce the false positive cases, such as threshold filtering with the fit score in the drug target prediction, significance analyzing with hypergeometric distribution approach in disease targets enrichment, and reasonable topologic parameter screening in the analyses of network (Wang et al., 2018). In addition, the combined prediction of multivariate data analyses and verification of pharmacologic tests confirmed the credibility of the model. The predicted results indicated that 14 constituents of SPN were interacted with 11 targets related to anemia in the network. As shown in Table 1, many candidate proteins were targeted by more than one compound. It suggested that these targets might play an important role in the hematopoiesis, the modulation of which could lead to the stimulation of various cytokines in the hematopoietic microenvironment, enhancement of the function of internal free radical scavenging system, facilitation of the absorption and utilization of iron, improvement of the bone marrow hematopoietic microenvironment, *etc*. (Wang et al., 2012b; Liu M. et al., 2014). The common cross-targets shared by multiple constituents implied that SPN might exert the synergistic therapeutic effect on anemia, which was probably more effective than single compound. This suggested that the herbal medicine might act on polypharmacological level, rather than on one specific protein in order to combat complex diseases, such as anemia.

**FIGURE 11:**
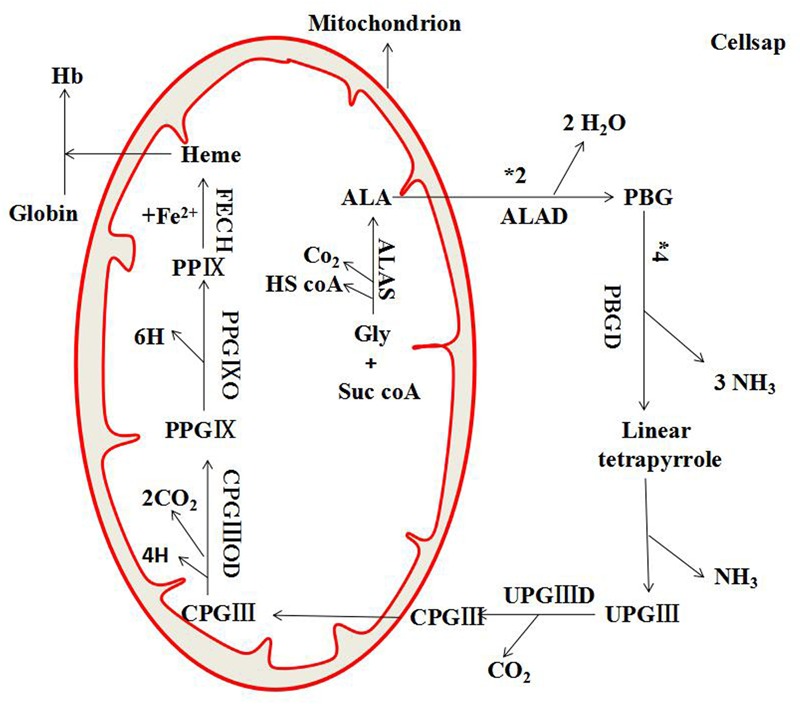
The metabolic pathway of FECH and heme. ALA, δ-aminolevulinic acid; ALAD, δ-aminolevulinic acid dehydrase; ALAS, δ-aminolevulinic acid synthase; CPGIII, coproporphyrinogen III; CPGIIIOD, coproporphyrinogen III oxidized decarboxylase; FECH, ferrochelatase; Gly, glycine; Hb, hemoglobin; PBG, porphobilinogen; PBGD, porphobilinogen deaminase; PPIX, protoporphyrinIX PPGIX, protoporphyrinogenIX; PPGIXO, protoporphyrinogenIX oxidase; Suc-CoA, succinyl–coenzyme A; UPGIX, uroporphyrinogen III; UPG?D, uroporphyrinogen III decarboxylase.

“Degree,” “betweenness centrality,” and “closeness centrality” are three key topological parameters that characterize the most influential nodes in a network. According to Li et al. (2007), if the “degree” of a node was more than twofolds of the median degree of all nodes in a network, such gene or protein was believed to play a critical role in the network structure, and it could be treated as a hub gene or a hub protein. “Betweenness centrality” was one of the significant indicators of network essentiality because proteins with high betweenness were essential for the functioning of the system by serving as a bridge of communication between several other proteins in the network (Melak and Gakkhar, 2015). And “closeness centrality” was another one of the significant indicators of network essentiality which represented the average length of the shortest paths to access all other proteins in the network. The higher the value, the more central the protein (Zhuang et al., 2015). Therefore, we used the above parameters to determine the importance of active constituents and action targets (the nodes in our network), as well as the extent of their influence on the spread of information through the network (Tang et al., 2015). Among the 11 predicted targets involved in the pathogenic process of anemia, FECH was shown relatively higher values of degree and closeness centrality, and was reported to be closely related to the production of heme. According to Figure 11, FECH is the terminal heme synthesis enzyme to catalyze the insertion of the imported iron into protoporphyrin IX to produce heme. Gene mutation in FECH may cause pathological changes like erythropoietic protoporphyria, an autosomal dominant disease which can develop into cholelithiasis and varying degrees of liver diseases (Casanova-González et al., 2010). It was reported that FECH forms an oligomeric complex with Mfrn1 and Abcb10 to synergistically integrate mitochondrial iron importation for heme biosynthesis (Chen et al., 2010). Since heme is an important raw material for hemoglobin synthesis and the increased heme level can resulted in a significant enhancement of human hemoglobin production (Liu L. et al., 2014), the variation of FECH and heme could be investigated to verify the hemopoiesis induced by active constituents of SPN.

From Table 1, the majority of compounds were linked with more than one target, indicating that these compounds might play the therapeutic effect by acting on multi-targets. Among the 14 compounds in our network, several of them might be essential. For example, in our previous work (Xiong et al., 2017a), we found the levels of some saponins in PN were increased along with the steaming time and temperature. Among them, ginsenosides Rh_4_, Rk_3_, 20(*R*)-Rg_3_, and 20(*R*)-Rh_1_ with higher contents or exclusively existed in SPN showed higher contributions to the activities of SPN. The result was consistent with the prediction in this research, that seven constituents of ginsenosides Rh_4_ (10), Rk_3_ (11), 20(*R*)-Rg_3_ (14), 20(*S*)-Rg_3_ (15), 20(*R*)-Rh_1_ (16), and 20(*S*)-Rh_1_ (17), and notoginsenoside R_2_ (20) were predicted to be interacting with the target of FECH for the treatment of anemia. It indicated that the network pharmacology approach had great potential to identify active constituents and alternative targets for the mechanism understanding and drug development of herbal medicines.

To further determine the active constituents, the analysis of fingerprint-effect relationship has been applied to screen characteristic constituents related to the hematinic effect of SPN. Multivariate data analyses such as PLSR and CCA are often used to specify a linear relationship between a set of dependent variables from a large set of independent variables, especially when the sample size is small relative to the dimension of these variables (Garza-Juárez et al., 2011; Wu et al., 2015). According to the results, Rk_3_ and 20(*S*)-Rg_3_ were predicted to be the major bioactive constituents of SPN treating anemia, which were also included in the prediction of network pharmacology analyses. The two constituents were reported to own various protective effects in previous studies. For example, 20(*S*)-Rg_3_ could prevent the progression of renal damage (Kang et al., 2010), protect against benzo[*a*]pyrene-induced genotoxicity in human cells (Poon et al., 2012), and protect against lipopolysaccharide-induced oxidative tissue injury in the liver of rats (Kang et al., 2009). And ginsenoside Rk_3_ was shown a protective effect against hypoxia-reoxygenation induced H9c2 cardiomyocytes damage, which was often used as a major ingredient of the compound preparation for ischemic heart diseases (Sun et al., 2013). To validate the predicted results, the effects of Rk_3_ and 20(*S*)-Rg_3_ on levels of blood routine parameters were investigated based on the BDS model. Compared to the model group, the high-dose Rk_3_ and 20(*S*)-Rg_3_ could significantly increase the levels of WBC, RBC, and PLT in a dose-dependent way. The high-dose 20(*S*)-Rg_3_ also made a significant difference on improving the content of Hb. It indicated that the two constituents had positive effect on improving the BDS of mice, which was consistent with the predicted results of network pharmacology and multivariate data analyses. Besides, the levels of FECH and heme could be increased by the treatment of Rk_3_ and 20(*S*)-Rg_3_ (Figure 10), suggesting that the two constituents exert the hematinic effect by regulating the predicted target and its downstream protein. Meanwhile, we noticed that the high-dose of ginsenosides Rk_3_ and 20(*S*)-Rg_3_ also inhibited the production of FECH and heme. That might be due to the bidirectional adjustment between FECH and heme that FECH was an integral factor for the biosynthesis of heme, whereas an excessive level of heme could inhibit the production of FECH (Dailey and Fleming, 1983; Wu et al., 2016). These results also indicated that the metabolism of FECH and heme was involved in the development of anemia, of which the stabilization could be regulated by SPN to resist hemolysis. However, further studies are needed to understand the precise nature of these contributing factors.

## Conclusion

To unveil the bioactive constituents and investigate the action mechanism of SPN for improving BDS, the network pharmacology approach coupled with multivariate data analyses were performed. In this study, we firstly predicted the active constituents and potential targets of SPN related to the treatment of anemia disease. The results showed that ginsenosides Rk_3_ and 20(*S*)-Rg_3_ were active constituents related to the hematinic effect of SPN, which acted on the targets of FECH and heme to improve the BDS. Although there could be various pathogens causing the incident of anemia and only the hemolytic type was investigated in the research, it also indicated potential areas for further research of SPN as a botanical remedy for the treatment of related diseases. The strategy employed does not only provide new insights for a deeper understanding of the chemical basis and pharmacology of SPN, but also demonstrate an efficient method for potential discovery of drugs originating from herbal medicines. Additional study on the therapeutic effect of SPN on other types of anemia and the involved pathways will be further carried out.

## Author Contributions

YX wrote this paper and carried out parts of data analyses. YH constructed the network and verified the predicted targets. LC did the multivariate data analyses and parts of pharmacologic tests. ZZ and YZ conducted parts of the pharmacologic tests. MN provided the technical support of network pharmacology. YX and XC supervised the project. All authors read and approved the final manuscript.

## Conflict of Interest Statement

The authors declare that the research was conducted in the absence of any commercial or financial relationships that could be construed as a potential conflict of interest.

## Footnotes

1 http://lilab.ecust.edu.cn/pharmmapper/index.php

2 https://david.ncifcrf.gov/

## Abbreviations

APH: acetylphenylhydrazine
BDS: blood deficiency syndrome
CCA: canonical correlation analysis
CTX: cyclophosphamide
FECH: ferrochelatase
FEJ: *Fufang E’jiao Jiang*
Hb: hemoglobin
OMIM: Online Mendelian Inheritance in Man
PLSR: partial least squares regression
PLT: platelet
PN: *Panax notoginseng*
PPI: protein–protein interaction
RBC: red blood cells
SPN: steamed *Panax notoginseng*
TCM: traditional Chinese medicine
TDT: TCM Database@Taiwan
WBC: white blood cells
