# Supplementary material for: Unveiling Active Constituents and Potential Targets Related to the Hematinic Effect of Steamed Panax notoginseng Using Network Pharmacology Coupled With Multivariate Data Analyses
Source: Front Pharmacol. 2019 Jan 8;9:1514. doi: 10.3389/fphar.2018.01514 (PMC6331451; doi:10.3389/fphar.2018.01514)
Supplement: Supplementary file 1 [file Data_Sheet_1.docx]

Supplementary Tables

Unveiling active constituents and potential targets related to the hematinic effect of steamed *Panax notoginseng* using network pharmacology coupled with multivariate data analyses

**Yin Xiong ^1,2,3,a,*^, Yupiao Hu ^1,a^, Lijuan Chen ^1,a^, Zejun Zhang ^1^, Yiming Zhang ^1^, Ming Niu ^4,*^,Xiuming Cui ^1,2,3,*^**

^1^ *Faculty of Life Science and Technology, Kunming University of Science and Technology, Kunming 650500, China*

^2^ *Yunnan Key Laboratory of Panax notoginseng,* *Kunming 650500, China*

^3^ *Laboratory of Sustainable Utilization of Panax notoginseng Resources, State Administration of Traditional Chinese Medicine, Kunming 650500, China*

^4^ *China Military Institute of Chinese Materia Medica, 302 Military Hospital of China, Beijing 100039, China*

* Correspondence:

Yin Xiong: [yhsiung@163.com](mailto:yhsiung@163.com)

Ming Niu: [nmbright@163.com](mailto:nmbright@163.com)

Xiuming Cui: [sanqi37@vip.sina.com](mailto:sanqi37@vip.sina.com)

**Table S1** **The sample information and peak area of fifteen common peaks in 18 batches of *Panax notoginseng* (PN) (Xiong et al., 2017a)**

| Sample | NO. | Peak area of each peak | | | | | | | | | | | | | | |
| --- | --- | --- | --- | --- | --- | --- | --- | --- | --- | --- | --- | --- | --- | --- | --- | --- |
|  |  | 1^a^ | 2 | 3 | 4 | 5 | 6 | 7 | 8 | 9 | 10 | 11 | 12 | 13 | 14 | 15 |
| Raw PN 1 | S1 | 360.8 | 1348.8 | 98.2 | 93.2 | 85.7 | 727.1 | 229.3 | 59.7 | 0 | 0 | 0 | 0 | 0 | 0 | 0 |
| Raw PN 2 | S2 | 337.5 | 1304.7 | 94.2 | 89.6 | 84.1 | 427.8 | 262.3 | 24.8 | 0 | 0 | 0 | 0 | 0 | 0 | 0 |
| Raw PN 3 | S3 | 560.6 | 1435.1 | 108.4 | 173.2 | 173.8 | 1356.8 | 462.3 | 24.8 | 0 | 0 | 0 | 0 | 0 | 0 | 0 |
| 105℃-2h | S4 | 430.4 | 1347.6 | 68.5 | 109.1 | 161 | 828.6 | 246.5 | 42.1 | 29.2 | 66.1 | 133.1 | 0 | 0 | 0 | 56.6 |
| 105℃-4h | S5 | 321.5 | 1182.1 | 134.8 | 121.9 | 191.9 | 759.7 | 234.3 | 59.9 | 101.9 | 167.4 | 252.2 | 38.7 | 0 | 49.3 | 108.2 |
| 105℃-6h | S6 | 251.4 | 1003.3 | 94.8 | 113.6 | 221.1 | 831.5 | 290.5 | 97.4 | 106.9 | 266.1 | 423.1 | 76.6 | 38.9 | 58.5 | 215.1 |
| 105℃-8h | S7 | 267 | 789.5 | 69.4 | 143.9 | 300.5 | 566.7 | 185 | 66.3 | 221.9 | 398.7 | 609 | 87.1 | 39.6 | 79.4 | 283.4 |
| 105℃-10h | S8 | 199.8 | 685.2 | 78 | 147.8 | 341.9 | 704.8 | 226.3 | 92.2 | 230.8 | 465.1 | 709.7 | 125.1 | 53.4 | 110.9 | 373.6 |
| 110℃-2h | S9 | 282.9 | 1160 | 109.7 | 104.3 | 176.8 | 821.1 | 232.6 | 93.6 | 57.7 | 153.1 | 266 | 35.1 | 0 | 0 | 102 |
| 110℃-4h | S10 | 201.4 | 1021.8 | 74.8 | 85.5 | 22.9 | 687.6 | 207.5 | 99 | 97.5 | 308.5 | 521.9 | 87.8 | 31.6 | 0 | 229 |
| 110℃-6h | S11 | 172.9 | 781 | 71.6 | 132 | 402.8 | 634.5 | 194.2 | 68.5 | 201.5 | 581.5 | 873.3 | 136.5 | 56.5 | 185.1 | 418.4 |
| 110℃-8h | S12 | 89.7 | 369.4 | 0 | 145.1 | 422.8 | 440 | 136.6 | 37.3 | 293.5 | 761.2 | 1136.9 | 173.9 | 79.8 | 256.9 | 559.7 |
| 110℃-10h | S13 | 0 | 164.4 | 0 | 149.9 | 550.2 | 415.5 | 115.3 | 21.9 | 314.8 | 892.8 | 1373.8 | 274.5 | 149.2 | 346.9 | 759.4 |
| 120℃-2h | S14 | 127.3 | 629.9 | 79.4 | 106 | 349.1 | 505 | 161.8 | 27.5 | 169.8 | 571.1 | 800.2 | 134.9 | 62.9 | 200.8 | 421 |
| 120℃-4h | S15 | 0 | 168.2 | 0 | 190.3 | 426.7 | 407 | 133.7 | 31.3 | 477.8 | 821.6 | 1281.5 | 142.5 | 133.7 | 444.3 | 939.3 |
| 120℃-6h | S16 | 0 | 0 | 0 | 110.3 | 474 | 249.1 | 52.8 | 0 | 331 | 1030.7 | 1641.7 | 279.6 | 135.6 | 709.8 | 1012.9 |
| 120℃-8h | S17 | 0 | 0 | 0 | 110.8 | 409.4 | 174.4 | 44 | 0 | 371.9 | 1002.8 | 1557.7 | 316.4 | 153.4 | 642.1 | 1052.2 |
| 120℃-10h | S18 | 0 | 0 | 0 | 97.1 | 230.6 | 196.8 | 0 | 0 | 368.3 | 1058.7 | 1748.3 | 293.5 | 150.7 | 532.3 | 1056.4 |
| C.V.^b^ (%) | | 81.1 | 67.4 | 75.5 | 23.1 | 52.4 | 46.6 | 53.9 | 70.2 | 76.7 | 77.6 | 78.0 | 85.6 | 95.6 | 114.7 | 88.8 |

^a^ Peaks 1-15,

^b^ C.V. (%) = *δ* / *μ* × 100; *δ* is the standard deviation, *μ* is the average value of peak are.

**Table S2**  **Factor cumulative explained variance of PLS**

| Principal component | *X* variable | Cumulative *X* variable | *Y* variable | Cumulative *Y* variable | *Q^2^*/cum | *R^2^* |
| --- | --- | --- | --- | --- | --- | --- |
| 1 | 0.693 | 0.693 | 0.512 | 0.512 | 0.625 | 0.664 |
| 2 | 0.100 | 0.793 | 0.236 | 0.748 | 0.756 | 0.757 |
| 3 | 0.053 | 0.846 | 0.106 | 0.854 | 0.782 | 0.622 |
| 4 | 0.018 | 0.864 | 0.017 | 0.871 | 0.815 | 0.837 |
